# The Relationship Between Thirst and the Incidence of Delirium in Patients Admitted to Intensive Care Units: A Cross Sectional Study

**DOI:** 10.1002/hsr2.71485

**Published:** 2025-11-27

**Authors:** Nasibeh Barani, Alun C. Jackson, Farshad Sharifi, Fatemeh Bahramnezhad

**Affiliations:** ^1^ Department of Critical Care Nursing, School of Nursing and Midwifery Tehran University of Medical Sciences Tehran Iran; ^2^ Centre on Behavioral Health University of Hong Kong Pokfulam Hong Kong SAR China; ^3^ Endocrinology and Metabolism Research Center, Clinical Sciences Institute, Endocrinology and Metabolism Research Institute Tehran University of Medical Sciences Tehran Iran; ^4^ Nursing and Midwifery Care Research Center, School of Nursing and Midwifery Tehran University of Medical Sciences Tehran Iran

**Keywords:** dehydration, delirium, intensive care unit, nursing, thirst

## Abstract

**Background and Aims:**

Delirium is an acute cognitive fluctuation commonly observed in intensive care units (ICUs) and influenced by multiple factors. Identifying these factors is crucial for effective prevention and management. This study aimed to examine the relationship between thirst and the incidence of delirium in adult ICU patients.

**Methods:**

This cross‐sectional study was conducted in 2022 on adult patients admitted to ICUs of hospitals affiliated with Kurdistan University of Medical Sciences, Iran. Thirst intensity was assessed using the validated Visual Analogue Scale (VAS), sedation/agitation by Richmond Agitation‐Sedation Scale (RASS), and delirium incidence was measured by the Confusion Assessment Method for the ICU (CAM‐ICU).

**Results:**

A total of 118 patients participated (59% male; mean age 46 years). Delirium was observed in 22.43% of patients. Thirst intensity was reported as mild by 51.69%, moderate by 29.66%, and severe by 18.64%. Delirium was significantly associated with increased thirst intensity, with moderate and severe thirst increasing the risk of delirium by approximately 4 and 14 times, respectively. Additionally, dehydration Statistical significant increased delirium risk by more than 10 times. Statistical significant associations were also found between delirium and biochemical markers such as blood urea nitrogen (BUN), creatinine (Cr), sodium, and plasma osmolality.

**Conclusion:**

ICU nurses should systematically assess and document key delirium risk factors, especially thirst, using validated tools such as VAS. Evidence‐based nonpharmacological strategies should be implemented for delirium prevention and management. Nurses must closely monitor laboratory parameters including BUN/Cr ratio, serum sodium, and plasma osmolarity, and promptly report abnormalities to physicians. Correction of hypernatremia or hyperosmolarity should be done under physician supervision; nurses facilitate early recognition and medical intervention but do not independently administer electrolyte‐correcting treatments.

## 1 Introduction

Patients admitted to the ICU deal with multiple stressors, such as 24‐h lighting with aggressive measures and sleep deprivation, pain, bed restraint due to multiple catheters, lack of privacy, being away from their family, inactivity, and thirst [1, 2], deal with fundamental problems, such as delirium [3], muscle wasting [4], malnutrition, immune dysfunction, severe and systemic infections, organ dysfunction and death [5, 6].

Among these factors, thirst is a common complaint, and a source of distress for many patients admitted to the ICU. Basically, thirst is perceived as an annoying feeling that results in a complex interaction between physiological homeostatic mechanisms and behavioral effects, leading to drinking in different situations. ICU patients usually experience some severe thirst distress during ICU admission [7]. Nelson et al. reported that of 50 patients admitted to ICU, 36 were able to speak, and of these 36. 9% reported high levels of thirst [8]. Similarly, Li et al. reported that thirst was severe in 40% of ICU patients who were on ventilators for an average of 6 days, with 30% of them experiencing it with moderate severity [9]. Whatever the cause of thirst, its destructive effects are clinically significant. Thirst and dehydration with direct effects on the gastrointestinal tract [10], kidneys [11], and cardiovascular system [12] cause many problems in patients admitted to the ICU. In addition, thirst as a sleep stressor causes problems such as insomnia, anxiety, restlessness, and depression [13] which can be precursors of important risk factors for delirium. In another study, failure to quench the thirst for more than 24 h was reported as a risk factor for delirium [7].

Delirium is an acute, multifactorial condition of confusion that is also accompanied by disturbances of consciousness and cognition. It is extremely common in the intensive care unit (ICU), with rates of incidence between 19% and 87%; it is particularly heightened in patients undergoing mechanical ventilation. [14]. Delirium has recently been defined in the Diagnostic and Statistical Manual of Mental Disorders(DSM‐5) as an acute disorder of attention, awareness, and cognitive impairment that is not explained by a pre‐existing neurocognitive disorder and is caused by another medical conditio [15, 16]. Delirium is a common clinical syndrome in hospitalized patients, especially in ICUs [17, 18], affecting approximately one‐third of ICU patient [19]. The prevalence of delirium varies from 9.2% in non‐ventilated surgical ICU patients [20] to 91% in mechanically ventilated cancer patients [21]. A delirium attack can cause a series of events that lead to permanent cognitive impairment, impaired performance, and other nosocomial complications, such as falls, fractures, prolonged hospital stays, and even death [15, 16].

Delirium is a multifactorial phenomenon. Nonpharmacological interventions play a significant role in controlling delirium symptoms, and pharmacological treatment is only used when delirium‐related agitation poses a risk [21]. However, there is still no globally accepted strategy for managing delirium, and various approaches are employed in this regard [22, 23]. One of the most important interventions is enhancing effective communication with patients experiencing delirium, which can be challenging due to fluctuations in mental status and concentration difficulties. Nurses and physicians should provide orientation information and use short, simple sentences [24]. Utilizing a translator, avoiding sudden movements, and ensuring the availability of glasses and hearing aids can help improve communication and reduce delirium symptoms [21]. Additionally, the presence of family and friends may contribute to patient comfort and support [25].

Environmental strategies can also be beneficial in managing delirium. Reducing noise levels, using large clocks and calendars, and maintaining appropriate lighting for day and night are measures that can improve patients' conditions [25]. The “timelessness” caused by windowless environments can disrupt the sleep‐wake cycle; therefore, adjusting lighting according to the time of day is recommended [26]. Moreover, physical restraints should be avoided, as they may exacerbate delirium and increase complications such as pressure ulcers and reduced mobility [25].

However, in cases where severe agitation threatens the safety of the patient or others, pharmacological intervention may be necessary. Despite recommendations for limited medication use, evidence suggests that pharmacological treatments remain widely employed, which may be due to a lack of staff training in nonpharmacological management strategies [27].

Although few studies have been performed on the relationship between thirst and delirium, some studies have identified dehydration, which usually occurs as thirst, as one of the factors that accelerate and facilitate delirium [28]. The role of dehydration in the pathogenesis of delirium is not fully understood, but factors such as tissue hypoperfusion (especially in the brain and kidneys), increased concentrations of drugs or their metabolites in reduced intravascular volumes, and decreased renal function in excretion and/or metabolism of drugs are involved in the development of delirium [28, 29].

Given that the onset of delirium may prolong hospitalization and contribute to mortality, and considering that approximately 40% of delirium cases are preventable, the implementation of appropriate strategies to reduce the risk of delirium is essential [29].

The aim of this study was to determine the relationship between thirst and the incidence of delirium in patients admitted to the ICU.

## 2 Methods

### 2.1 Design

This cross‐sectional study was conducted in Kurdistan Province, Iran, from April 2022 to December 2022. The study was reported following the Strengthening the Reporting of Observational Studies in Epidemiology (STROBE) guidelines [30].

### 2.2 Setting

In Sanandaj city in the Kurdistan Province, the study was conducted in the ICUs of Ba'ath, Kowsar, and Tawhid hospitals, which are all affiliated with Kurdistan University of Medical Sciences. Kowsar Hospital had four ICUs with a bed capacity of 40. Ba'ath Hospital had one 10‐bed ICU for female patients, and Tawhid Hospital had two ICUs with 21 beds. The patient‐to‐nurse ratio in these ICUs was 2:1. All ICUs were general ICUs and were not subspecialized.

The task‐based model of nursing structure was utilized in these hospitals, where nurses performed tasks by shift duties and responsibilities rather than patient continuity. ICU nurses were educated to at least a bachelor's level in nursing, and most had additional in‐service education and critical care certifications such as airway management, hemodynamic monitoring, and delirium screening.

Though protocol‐driven, systematic delirium and thirst management was not formally integrated into the ICU nursing care models, nursing staff made it a regular practice to assess consciousness level, hydration, and implement non pharmacologic interventions such as oral hygiene regularly, maintaining communication, reorientation, limiting noise and light during night, and encouraging early mobilization as possible. Nurses also contributed to delirium screening via CAM‐ICU and assisted in identifying at‐risk patients for dehydration or hypernatremia and thus could initiate early medical intervention.

### 2.3 Participants and Sample Size

The statistical population included ICU patients. Using simple random sampling, 118 eligible patients were selected. Inclusion criteria were: willingness to participate, age between 18 and 60 years, ICU stay over 24 h, RASS score between −1 and +1 before thirst assessment, and being under mechanical ventilation (via endotracheal tube or tracheostomy). Exclusion criteria included a history of dementia, oral surgery, alcohol or drug abuse, sensory deficits (deafness, blindness, aphasia), the need for sedatives or opioids, or discharge before completion of the study.

### 2.4 Data Collection

After obtaining ethical approvals from Tehran and Kurdistan Universities of Medical Sciences, informed written consent was obtained from patients or their legal guardians. Assessments of thirst and delirium were conducted between 4 PM and 8 PM. Data collection was conducted by the researcher (the first author). The following tools were used for data collection:

#### 2.4.1 Visual Analog Scale (VAS)

The VAS was used to assess self‐reported thirst intensity. The instrument's validity was supported by Langley and Sheppeard [31] and used in Iranian populations by Mazlom et al. [32]. Content validity was reviewed by 10 faculty members and ICU fellows. Reliability testing in 20 conscious ICU patients showed a coefficient of 0.89. The scale ranges from 0 (no thirst) to 10 (severe thirst). Thirst intensity was classified as mild (0–3), moderate (4–6), and severe (7–10) [33].

#### 2.4.2 Confusion Assessment Method for the ICU (CAM‐ICU)

This validated screening tool consists of four items. Delirium is diagnosed when the first two criteria are positive, along with at least one of the remaining two. Originally validated in 1990, and further studied by Thomason et al. (2005) [34]. In Iran, it was validated in open‐heart surgery patients by Zolfaghari et al. (2012) with *r* = 0.94 and sensitivity of 76.6% [35].

#### 2.4.3 Plasma Osmolality and Laboratory Tests

Routine blood samples were taken daily, but for consistency, only results from even days were recorded. Thirst was also assessed using biochemical markers: BUN ≥ 6.7 mmol/L, creatinine ≥ 150 µmol/L, BUN/Cr ratio > 18, sodium > 149 mmol/L, and plasma osmolality > 295 mOsm/kg [36, 37].

Plasma osmolality was calculated using the formula [38]:

Plasmaosmolality=2×sodium(mmol/L)+BUN(mg/dL)/2.8+glucose(mg/dL)/18

* BUN (Blood Urea Nitrogen) is a medical test that measures the amount of nitrogen in the blood that comes from the waste product urea. Urea is formed in the liver when the body breaks down proteins and is normally filtered out by the kidneys. Elevated BUN levels can indicate impaired kidney function, dehydration, or other medical conditions.

### 2.5 Data Analysis

Data were analyzed using statistical software for data science (STATA) v12. Statistical significance was set at *p* < 0.05. Descriptive statistics included means, standard deviations, and interquartile ranges. Categorical variables were reported as frequencies. Logistic regression, Poisson regression, and Cox models were used to evaluate factors influencing delirium. Sensitivity analysis was done for missing data. Patients were categorized based on presence or absence of thirst (for analysis purposes only). All 118 patients completed the VAS and CAM‐ICU assessments without attrition.

### 2.6 Ethical Considerations

All procedures followed national/institutional ethical standards and the Helsinki Declaration. Approval was granted by the Institutional Review Board (IRB) of Tehran University of Medical Sciences (Ethics code: IR.TUMS.FNM.REC.1399.112). Written informed consent was obtained from all participants or legal representatives. For illiterate patients, the form was explained and signed with a fingerprint.

## 3 Results

A total of 118 patients were included in the study, of whom 59% were male. The mean age of the participants was 46.0 years (SD = 0.697). Additional demographic characteristics are presented in Table 1. Regarding clinical symptoms, 95.76% of patients reported no sleep disorders, 94.92% had no vomiting, 74.58% had no history of diuretic use, and 95.32% did not report dyspnea.

**Table 1:** Demographic characteristics of patients.

Variable	Category	Frequency (*n*)	%
Gender	Male	70	59.32
	Female	48	40.68
Age (year)	18–29	18	15.25
	30–39	22	18.64
	40–49	23	19.49
	50–60	55	46.61
BMI (body mass index)	< 18.5	2	1.69
	18.5–24.9	70	59.32
	25–29.9	38	30.20
	30–34.9	8	6.78
Marital status	Married	93	78.81
	Single	18	15.25
	Widow	5	4.24
	Divorced	2	1.69
Education	Illiterate	34	28.81
	Below diploma	39	33.05
	Diploma	29	24.58
	Associate degree	16	13.56
Living with family	Yes	112	95.73
	No	5	4.27
Reason for hospitalization	Trauma	37	31.36
	Ischemic CVA	12	10.17
	Hemorrhagic CVA	12	10.17
	Being stabbed	4	3.39
	Gynecology	9	7.63
	Cancer	10	8.47
	Others	34	28.81^a^
Underlying diseases	Diabetes	4	5.26
	Hypertension	11	14.47
	Anemia	1	1.32
	Kidney disease	10	13.16
	No underlying disease	14	18.24
	Total number of underlying diseases	36	47.37

^a^ Cerebrovascular Accident (CVA).

Among the 118 patients, 51 (43.22%) were diagnosed with delirium according to CAM‐ICU criteria, while 67 (56.78%) did not meet the criteria for delirium. The mean RASS score was −0.17, and the average thirst intensity measured by the VAS scale was 3.76. The mean body temperature was 36.92°C, oxygen saturation was 96.17%, systolic blood pressure was 126.10 mmHg, and heart rate was 85.02 beats per minute. Laboratory findings included a mean serum sodium level of 140.64 mmol/L, a mean BUN of 16.57 mg/dL, and a mean plasma osmolality of 292.76 mOsm/kg (Table 2).

**Table 2:** Clinical characteristics of the study participants.

Variable	Mean	Standard deviation	Min	Max
VAS^a^ for thirst	3.76	3.21	0	9.75
RASS^b^	−0.17	0.55	−1	1
Body temperature (°C)	36.92	0.83	36.17	45.6
Oxygen saturation (%)	96.17	3.47	33.67	99.5
Systolic blood pressure (mmHg)	126.10	17.23	91.5	160
Diastolic blood pressure (mmHg)	78.47	10.28	60	103.33
Heart rate (beat/min)	85.02	11.39	64	114.83
Sodium (mEq/L)	140.64	3.41	133.5	151.75
BUN^c^ (mg/dL)	16.57	6.30	7.66	45.5
Mean ratio of urea to creatinine	15.64	4.72	8.07	30.68
Plasma osmolarity (mOsm/kg)	292.76	11.90	222.33	330.75

^a^ Visual analog scale.

^b^ Richmond agitation‐sedation scale.

^c^ Blood urea nitrogen.

Logistic regression analysis showed that the odds ratio (OR) for BUN was 1.10 (95% CI: 0.989–1.22; *p* = 0.07), for creatinine was 0.42 (95% CI: 0.094–1.93; *p* = 0.26), and for sodium was 1.30 (95% CI: 1.10–1.53; *p* < 0.001). The OR for the BUN/creatinine ratio was 10.14 (95% CI: 2.57–39.94; *p* < 0.001), and for plasma osmolality was 5.11 (95% CI: 1.90–13.73; *p* < 0.001) (Table 3).

**Table 3:** Relationship between serum BUN and creatinine, sodium, BUN/Cr ratio, plasma osmolality, and delirium.

Variable	Odds ratio (OR)	*p* value	95% Confidence interval (CI)
BUN^a^	1.10	0.07	0.989 – 1.22
Creatinine	0.42	0.26	0.094 – 1.93
Sodium (per unit increase)	1.30	< 0.001	1.10 – 1.53
BUN/Creatinine ratio (dehydration)	10.14	0.000	2.57 – 39.94
Plasma osmolarity	5.11	< 0.001	1.90 – 13.73

^a^ Blood urea nitrogen.

Regarding secondary outcomes, 83.90% of the patients were not dehydrated based on the BUN/creatinine ratio. In addition, 88.98% had serum sodium levels within the normal range (< 149 mmol/L), and 53.39% had normal plasma osmolality values.

Multivariate logistic regression revealed a significant association between thirst intensity (measured using VAS) and the presence of delirium. The frequency of delirium differed significantly according to categorized levels of thirst intensity, as presented in Table 4. Moreover, the Poisson regression model indicated that the incidence rate of delirium varied depending on the duration of moderate to severe thirst. Table 5 summarizes the distribution of delirium incidence across different intervals of thirst duration.

**Table 4:** The relationship between thirst and delirium in patients participating in the study.

Variable	Odds ratio (OR)	*p* value	95% CI
Moderate intensity of thirst (VAS)^a^	4.31	0.01	1.34–13.80
Severe intensity of thirst	14.11	0.000	2.90–68.28

^a^ Visual analogue scale.

**Table 5:** The relationship between the numbers of delirium occurrences in the presence of moderate to severe thirst.

**Variable**	**IRR** a	*p* value	**95% CI** b
Exposure to moderate to severe thirst for 1–6 days	1.68	0.000	1.01–2.78
Exposure to moderate to severe thirst for 7–12 days	3.75	0.000	2.30–6.11

^a^ Incidence rate ratio.

^b^ Confidence interval.

## 4 Discussion

In this study of 118 ICU patients, the mean age was 46 years, and 59% were male. Most patients reported no major symptoms such as vomiting (94.92%), sleep disorder (95.76%), or dyspnea (95.32%), and 74.58% did not receive diuretics. These demographic and clinical characteristics are important when interpreting the results, as they influence vulnerability to complications such as thirst and delirium in critically ill patients.

Regarding the primary outcome, our findings revealed a significant relationship between thirst intensity and the occurrence of delirium. Moderate thirst increased the risk of delirium by more than four times, while severe thirst increased it by over 14 times. Furthermore, the risk of delirium in patients with moderate to severe thirst lasting 1 to 6 days increased by 68%, and in cases where thirst persisted for more than 6 days, the risk of delirium tripled. These findings are consistent with the study of Sato et al. [7], which highlighted similar associations between thirst and cognitive disturbances.

In this study, delirium was assessed using the CAM‐ICU tool, while Sato et al. used the Intensive Care Delirium Screening Checklist (ICDSC) for assessment. Both tools are validated for ICU settings, but differ in diagnostic criteria and methodology. CAM‐ICU is widely used for its sensitivity and specificity in non‐verbal ICU patients [34]. Despite methodological differences, both tools have demonstrated utility in identifying delirium early, facilitating prompt intervention. In Sato's study, daily screening was performed, aligning with best practices. Our findings emphasize the importance of systematic delirium monitoring using standardized tools like CAM‐ICU to reduce cognitive complications in ICU settings.

Moving to secondary outcomes, while BUN and creatinine alone were not significantly associated with delirium (*p* = 0.07 and *p* = 0.26, respectively), the BUN to creatinine ratio—a marker for dehydration—showed a strong association with increased risk of delirium (OR = 10.14, 95% CI: 2.57–39.94, *p* = 0.000). These results are consistent with Foroughan et al. (2020) [39] and Iwata et al. (2020) [40], who also demonstrated the link between dehydration and delirium. Dehydration reduces cerebral perfusion, which may impair brain function and trigger delirium.

In addition, increased levels of sodium (OR = 1.30, 95% CI: 1.10–1.53, *p* < 0.001) and plasma osmolality (OR = 5.11, 95% CI: 1.90–13.73, *p* < 0.001) were significantly associated with delirium. These results align with the findings of Mariz et al. (2019) [41], who reported that elevated osmolality and hypernatremia contribute to delirium. In contrast, Wang et al. (2018) [42] and Santos et al. (2017) [43] reported differing outcomes, potentially due to variations in study populations, such as age, comorbidities, or care protocols.

In our setting, non‐pharmacologic prevention and treatment of delirium are prioritized. These include regular delirium assessments, oral care, noise/light control, hydration monitoring, early mobilization, and family engagement. Nurses play a key role in these processes, supported by protocols and in‐service training [35]. Pharmacologic interventions are used conservatively and only in severe cases, adhering to current ICU guidelines.

The ICU environment itself also contributes to cognitive outcomes. Environmental adjustments (e.g., noise reduction, proper lighting, air quality), reorientation techniques, and communication tools (e.g., glasses, hearing aids) are routinely used to reduce cognitive stress. These findings highlight the multifactorial nature of delirium and reinforce the need for comprehensive management strategies in critically ill patients. Also, it is possible to assess delirium once per day, but it is recommended to do that three times per day on ICU [44].

### 4.1 Strength and Limitations

This study benefits from several key strengths. Firstly, it involved a large sample size of 118 patients, which strengthens the statistical power and reliability of the findings. The study also focused on nonpharmacological interventions for delirium, aligning with current guidelines that emphasize minimizing pharmacological treatments. Validated tools such as CAM‐ICU and VAS were used for assessment, ensuring the reliability of the data. Furthermore, data collection was thorough and consistent, conducted by a single researcher to reduce variability. Ethical considerations were rigorously followed, with informed consent obtained from patients or their legal guardians. Additionally, a multidisciplinary approach involving healthcare professionals from various fields contributed to a comprehensive management strategy for ICU patients. One of the most significant strengths of our study is that we measured serum osmolality. Moreover, we conducted follow‐ups on patients for delirium rather than assessing them only once. Furthermore, we collected samples from multiple hospitals, enhancing the study's diversity and applicability. However, this study has several limitations. Despite the relatively large sample size, it remains small in comparison to broader population studies, and the cross‐sectional nature of the research limits causal interpretations. Cultural, linguistic, and contextual factors may have influenced the findings, making generalization challenging. Additionally, the use of simple random sampling may not have ensured full representativeness of the target population. Another limitation is the lack of disease severity assessment using the acute physiology and chronic health evaluation (APACHE) system, which could have significantly impacted the analysis. Furthermore, thirst and delirium were measured only once per day, whereas international recommendations suggest assessing these parameters up to three times per day. This limitation may have led to an underestimation of fluctuations in these conditions.

## 5 Conclusion

This study aimed to investigate the relationship between thirst and delirium in ICU patients at Kurdistan University of Medical Sciences hospitals in 2021. Using VAS for thirst and CAM‐ICU tools, 118 patients were assessed. Findings revealed a significant correlation between thirst intensity, dehydration markers (BUN/creatinine ratio, sodium levels, plasma osmolarity), and delirium incidence. Given that delirium is largely preventable, regular thirst assessment, hydration management, and monitoring of dehydration markers can aid in its prevention. The study highlights the need for nurses to address patient thirst in ICUs and suggests further research to develop effective management strategies. Due to time and resource constraints, a pilot study was not conducted; however, it is recommended for future research to enhance sample representativeness.

## Author Contributions

**Nasibeh Barani:** conceptualization, writing – original draft, data curation, investigation. **Alun C Jackson:** conceptualization, methodology, validation, investigation, writing – review and editing, writing – original draft. **Farshad Sharifi:** methodology, formal analysis, software, writing – original draft. **Fatemeh Bahramnezhad:** data curation, supervision, conceptualization, writing – review and editing, writing – original draft.

## Conflicts of Interest

The authors declare no conflicts of interest.

## Transparency Statement

The lead author, Fatemeh Bahramnezhad, confirms that this manuscript represents an honest, accurate, and transparent account of the study being reported. All relevant aspects of the study have been included, and any deviations from the original plan (if applicable) have been clearly explained. All authors have reviewed and approved the final version of the manuscript. The corresponding author had full access to all study data and accepts full responsibility for the integrity of the data and the accuracy of the data analysis.

## Data Availability

All data supporting the findings of this study are included within the article and its supporting materials. No additional datasets were generated or analyzed during the current study.
